# Childhood chronic conditions and health-related quality of life: Findings from a large population-based study

**DOI:** 10.1371/journal.pone.0178539

**Published:** 2017-06-02

**Authors:** Guannan Bai, Marieke Houben–van Herten, Jeanne M. Landgraf, Ida J. Korfage, Hein Raat

**Affiliations:** 1 Department of Public Health, Erasmus MC, University Medical Centre Rotterdam, Rotterdam, South Holland, the Netherlands; 2 Socio-economic and Spatial Statistics, Statistics Netherlands, Heerlen, Limburg, the Netherlands; 3 HealthActCHQ, Boston, Massachusetts, United States of America; University of Bremen, GERMANY

## Abstract

The objective of this study was to assess the impact of health-related quality of life (HRQOL) across prevalent chronic conditions, individually and comorbid, in school-aged children in the Netherlands. 5301 children aged 4–11 years from the Dutch Health Interview Survey were included. Parents completed questionnaires regarding child and parental characteristics. HRQOL of children was measured using the Child Health Questionnaire Parent Form 28 (CHQ-PF28). Independent-t tests were used to assess differences in the mean scores of the CHQ-PF28 summary scales and profile scales between children with a prevalent chronic condition (excluding or including children with multiple chronic conditions) and children without a chronic condition. Cohen’s effect sizes (d) were calculated to assess the clinical significance of difference. The mean age of children was 7.55 (SD 2.30) years; 50.0% were boys. In children without any chronic condition, the mean score of physical summary scale (PhS) was 58.53 (SD 4.28) and mean score of the psychosocial summary scale (PsS) was 53.86 (SD 5.87). Generally, PhS and/or PsS scores in children with only one condition were lower (p<0.05) than for children without chronic conditions. When children with multiple conditions were included, mean scores of CHQ-PF28 summary and profile scales were generally lower than when they were excluded. The present study shows important information regarding the impact of prevalent chronic conditions on HRQOL in a representative population-based sample of school-aged children in the Netherlands. The information could be used for developing a more holistic approach to patient care and a surveillance framework for health promotion.

## Introduction

Over the past decades, the prevalence of chronic conditions of children has increased over time [1, 2]. Particularly, asthma and behavioral problems (e.g. attention deficit/hyperactivity disorder) show a greater increase in prevalence [3–5]. Clinical studies have suggested that children with particular chronic conditions may experience impairments of health-related quality of life (HRQOL) [6–15]. HRQOL is a multidimensional concept that focuses on the individuals’ perceptions of their physical, psychological, and social functioning [16]. However, the generalization of findings is often restricted by the small sample size in the above studies. In recognition of this need, recently the associations of childhood chronic conditions with HRQOL have been assessed in representative population samples [17–23]. The chronic conditions evaluated in most of the above studies were selected based on experts’ opinions [20] or on clinical importance rather than prevalence in the population [19, 21, 22]. The association between prevalent chronic conditions of children and HRQOL is not clear. A relevant issue is comorbidity, which is also common in the child population. Only three studies evaluated the impact of comorbidity on children’s HRQOL [19–21] in population-based studies. Little is known about the profiles of child’s HRQOL across prevalent childhood chronic conditions in a representative population sample.

The present investigation used data embedded in the Dutch Health Interview Survey (DHIS) conducted from 2010 to 2013. The five most prevalent child chronic conditions were identified (asthma; eczema; dyslexia; ADHD; migraine/severe headache). The goal of the current investigation was to assess difference in HRQOL for children with only one of the five prevalent conditions (without co-morbidity) in comparison to children without any chronic condition. Difference in HRQOL for children with one of the five prevalent conditions including the presence of co-morbidity was also compared.

## Methods

### Participants and procedures

Data used for the current investigation was extrapolated from the Dutch Health Interview Survey (DHIS), conducted by Statistics Netherlands. DHIS is a cross-sectional survey amongst the Dutch population living in non-institutionalized households. Each month, a stratified two-step-sample of persons is taken from the Dutch Municipal Personal Records [23]. For this investigation, a four-year set of survey responses for children ages 4–11 years were used. Only one parent participated in the interview. Between January 2010 and December 2013, parents of 6499 children aged 4–11 years were interviewed. The yearly response rate for children ages 4–11 years is approximately 73%.

Parents received written study information from Statistic Netherlands and participation was elective. According to Dutch law (Wet medisch wetenschappelijk onderzoek met mensen), formal approval (e.g., from a medical ethics committee) was not required as this study relied on secondary anonymized data collection in the context of performing statutory tasks. Data collection and processing was in strict accordance with the national standard. At no time did the datasets contain direct identifiers [23].

### Measures

#### Chronic conditions

Parents were asked to indicate if their child had ever had cancer, or experienced other health or behavioral issues during the previous 12 months: congenital heart defect, diabetes, migraine/ severe headache, asthma, psoriasis, eczema, arthritis/rheumatism, severe/protracted disorders of the intestines, back, neck/shoulder, arm or hand; dyslexia, autism or conditions related to autism like Asperger’s, intellectual disability, and presence of at least three core ADHD symptoms (DSM-criteria: restless behavior/not being able to sit still, fidgeting/squirming, short attention span). An open-ended question about any other chronic conditions and behavioral issues not mentioned was also included. For each condition, possible responses were “no” (i.e. does not have the condition), “yes” (i.e. has the condition). For all chronic conditions except for ADHD, if the parent answered “yes”, a following question should be answered: “Has your child been seen by the family physician or medical specialist in the previous 12 months”.

To identify the five most prevalent in the Dutch child population, the data was weighted to take into account the person’s probability of selection [24]. By doing so, responses are adjusted to the actual distribution of persons in the target population, allowing generalization at the national level. The prevalent conditions in the Dutch child population in 2010–2013 according to this health survey were asthma (6.4%), eczema 5.8(%), dyslexia (5.1%), attention deficit/hyperactivity disorder (ADHD) (3.1%) and migraine/severe headache (2.7%) [25][26], followed by disorders of the intestines (1.7%), intellectual disability (1.2%), congenital heart disease (1.0%) and so on [27]. In the present study, we only focused on the five most prevalent chronic conditions.

#### Health-related quality of life

CHQ-PF28 is a 28-item, parent-reported measurement of children’s HRQOL. CHQ-PF 28 was selected because it has been rigorously translated into 78 languages (http://www.healthactchq.com/chq-t.php) and specifically evaluated for use in the Netherlands and it is easy to administer in large population studies [28–30]. Based on 13 scales, CHQ-PF28 measures the HRQOL of children and their families. The child’s HRQOL is measured by the following ten scales: Physical Functioning (PF)); Role/Social-Physical (RP); General Health Perception (GP); Bodily Pain (BP); Role/Social Emotional/Behavioral (REB); Self-Esteem (SE); Mental Health (MH); Behavior (BE); Parental Impact-Time (PT); and Parental Impact-Emotional (PE). These ten scales are involved into scoring the Physical Summary Component Scale (PhS) and the Psychosocial Summary Component Scale (PsS). Furthermore, there are Family Activities (FA), Family Cohesion (FC) and Change in Health (CH) scales. In the present study, data on the ‘Change in Health Scale’ was not reported. Items are responded on four-, five-, or six- Likert-type scales and then standardized on a 0–100 continuum. Higher scores represent better HRQOL. PhS and PsS are based on factor weights from a US representative sample of children aged 5–18 years of age [31]. A score of 50 represents the mean of the US reference population sample and the standard deviation is ten points above/below the mean. The weighted US values to derive two component summary scales have been used with success in both Dutch and other international studies [19, 20, 23, 28, 29]. In our study, the Dutch version of the parent-completed CHQ-PF28 was administered via the internet, or via a structured telephone or face-to-face interview as part of the larger DHIS interview [32].

#### Covariates

Data regarding children’s age, sex, ethnic background, body mass index, single parent family, number of acute health complaints and education level of the parent who completed the interview, which were considered as potential confounders, were collected by questionnaire during the interview. Acute health complaints in the present study are defined as the occurrence of headache, tiredness, back pains, muscle or joint pains during the last 14 days.

Parental education level (low, medium, high) and ethnic background of the child were defined according to the Dutch standards classifications [33][34]. Low education level includes pre-primary, primary and lower secondary education; medium education level is similar to upper secondary education; high education level includes bachelor and master degrees and doctorate. If there are two parents with a different education level, then the highest level is chosen. Children for whom at least one parent was born outside the Netherlands were identified as (second generation) immigrants (even if the child was born in the Netherlands). Western immigrants were classified as those originating from Europe (excluding Turkey), North America, Oceania, Indonesia or Japan; Non-western immigrants were classified as those originating from Africa, South America, Asia (excluding Indonesia and Japan) or Turkey.

### Statistical analyses

6499 parents of the same number of children were interviewed at enrollment. Children with ‘outliers’ (values above/below 3xSD +/- mean) regarding one of the two summary CHQ-PF28 scales were deleted (n = 252). Additionally, 122 children were excluded due to missing more ≥1 item on the CHQ-PF28. Also excluded were children with a reported condition that was not asthma, eczema, ADHD, dyslexia and severe headache (n = 430) or for whom >2 chronic conditions were reported (n = 394). Thus a final sample of 5301 children was used for data analyses. (see S1 Fig).

Mean and standard deviations of the CHQ-PF28 scale and summary scores were calculated for children without reported chronic conditions (n = 4539), and for children with one of the five prespecified chronic conditions (asthma, n = 235; eczema, n = 192; dyslexia, n = 207; ADHD, n = 51; and migraine/severe headache, n = 77). Independent sample t-tests were used to assess differences in the mean scores of the scales and summary scales between children with and without a chronic condition. The relevance of the difference was assessed using Cohen’s effect size. The difference in mean scores was divided by the largest SD and interpreted as (d): 0.2 ≤ d < 0.5 small difference, 0.5 ≤ d < 0.8 moderate, and d ≥ 0.8 large [35]. Additionally, taking into account the covariates, general linear models were applied to assess differences in the mean scores of scales and summary scales between the subgroups.

Independent sample t-tests were also applied to assess differences in the mean scores of CHQ-PF28 scales and summaries between children with and without a chronic condition when children with multiple conditions were included. Cohen’s effect size was used to assess the clinical relevance of the difference. Additionally, we assessed the differences in the CHQ-PF28 mean scores of the scales and summaries between children who were seen by the family physician or medical specialist in the previous 12 months and children who were not.

A p-value <0.05 was considered to be statistically significant. Analyses were performed using SPSS 22.0.

## Results

Table 1 presents the general characteristics of the population for analysis. There were 5301 children (2651 girls and 2650 boys) aged 4–11 years (mean: 7.55, standard deviation: 2.30). 19.4% of the children were non-Dutch; 11.1% from a single parent family; 32.2% children had one or more acute health complaints. Compared to children without any chronic condition, children with dyslexia were more often male, older, Dutch and had more acute health complaints; children with asthma and children with eczema more often lived in the single parent family; parents of children with migraine/severe headache more often reported low/medium education.

**Table 1:** General characteristics of study population (n = 5301).

Variables	Total (N = 5301)	No chronic condition (n = 4539)	Asthma (n = 235)	Eczema (n = 192)	ADHD (n = 51)	Dyslexia (n = 207)	Migraine/ severe headache (n = 77)
**Children**							
**Sex**							
**Male**	2650 (50.0)	2242 (49.4)	**141 (60.0)**	87 (45.3)	29 (56.9)	**118 (57.0)**	33 (42.9)
**Female**	2651 (50.0)	2297 (50.6)	**94 (40.0)**	105 (54.7)	22 (43.1)	**89 (43.0)**	44 (57.1)
**Age in years, mean (SD)** 7.55±2.30							
**4**	634 (12.0)	570 (12.6)	25 (10.6)	28 (14.6)	7 (13.7)	**1 (0.5)**	3 (3.9)
**5**	654 (12.3)	579 (12.8)	33 (14.0)	27 (14.1)	8 (15.7)	**3 (1.4)**	4 (5.2)
**6**	654 (12.3)	582 (12.8)	27 (11.5)	25 (13.0)	4 (7.8)	**7 (3.4)**	9 (11.7)
**7**	680 (12.8)	579 (12.8)	38 (16.2)	24 (12.5)	10 (19.6)	**18 (8.7)**	11 (14.3)
**8**	645 (12.2)	549 (12.1)	24 (10.2)	23 (12.0)	10 (19.6)	**26 (12.6)**	13 (16.9)
**9**	663 (12.5)	557 (12.3)	23 (9.8)	22 (11.5)	5 (9.8)	**46 (22.2)**	10 (13.0)
**10**	667 (12.6)	551 (12.1)	35 (14.9)	20 (10.4)	3 (5.9)	**46 (22.2)**	12 (15.6)
**11**	704 (13.3)	572 (12.6)	30 (12.8)	23 (12.0)	4 (7.8)	**60 (29.0)**	15 (19.5)
**Ethnic background**							
**Native Dutch**	4273 (80.6)	3655 (80.5)	177 (75.3)	142 (74.0)	46 (90.2)	**188 (90.8)**	65 (84.4)
**Immigrant, Western**	324 (6.1)	275 (6.1)	18 (7.7)	14 (7.3)	0 (0.0)	**14 (6.8)**	3 (3.9)
**Immigrant, Non-western**	704 (13.3)	609 (13.4)	40 (17.0)	36 (18.8)	5 (9.8)	**5 (2.4)**	9 (11.7)
**Body mass index**							
**Normal weight**	3755 (70.8)	3221 (71.0)	161 (68.5)	142 (74.0)	33 (64.7)	135 (65.2)	63 (81.8)
**Overweight**	397 (7.5)	323 (7.1)	28 (11.9)	18 (9.4)	3 (5.9)	20 (9.7)	5 (6.5)
**Obese**	113 (2.1)	93 (2.0)	5 (2.1)	6 (3.1)	4 (7.8)	4 (1.9)	1 (1.3)
**Unknown**	1036 (19.5)	902 (19.9)	41 (17.4)	26 (13.5)	11 (21.6)	48 (23.2)	8 (10.4)
**Family structure**							
**Two parent family**	4683 (88.3)	4049 (89.2)	**193 (82.1)**	**157 (81.8)**	43 (84.3)	181 (87.4)	**60 (77.9)**
**Single parent family**	590 (11.1)	466 (10.3)	**41 (17.4)**	**35 (18.2)**	7 (13.7)	25 (12.1)	**16 (20.8)**
**Other/Unknown**	28 (0.5)	24 (0.5)	**1 (0.4)**	**0 (0.0)**	1 (2.0)	1 (0.5)	**1 (1.3)**
**Number of acute health complaints**							
**None**	3596 (67.8)	3180 (70.1)	**140 (59.6)**	**104 (54.2)**	**25 (49.0)**	**133 (64.3)**	**14 (18.2)**
**1**	1139 (21.5)	927 (20.4)	**55 (23.4)**	**59 (30.7)**	**18 (35.3)**	**48 (23.2)**	**32 (41.6)**
**2**	450 (8.5)	356 (7.9)	**26 (11.1)**	**23 (12.0)**	**7 (13.7)**	**16 (7.7)**	**22 (28.6)**
**3 or more**	116 (2.2)	76 (1.7)	**14 (6.0)**	**6 (3.1)**	**1 (2.0)**	**10 (4.8)**	**9 (11.7)**
**Parental education level**							
**Low**	713 (13.4)	603 (13.3)	34 (14.5)	31 (16.1)	9 (17.6)	24 (11.6)	**12 (15.6)**
**Medium**	1689 (31.9)	1423 (31.4)	76 (32.3)	62 (32.3)	21 (41.2)	73 (35.3)	**34 (44.2)**
**High**	2230 (42.1)	1936 (42.7)	91 (38.7)	78 (40.6)	14 (27.5)	94 (45.4)	**17 (22.1)**
**Unknown**	669 (12.6)	577 (12.7)	34 (14.5)	21 (10.9)	7 (13.7)	16 (7.7)	**14 (18.2)**

Values are numbers and percentages. Bold print indicates significant difference (p<0.05) of children with only one condition (asthma, eczema, dyslexia, ADHD, or migraine/severe headache) compared with children without chronic conditions.

Mean scores of CHQ-PF28 scales and summaries in children with a specific condition (asthma, eczema, dyslexia, ADHD, migraine/severe headache) were lower than children without any chronic condition (see Table 2). Regarding the summary scales, children with only asthma were reported with a relatively lower mean score of the physical summary scale than children without any chronic condition (54.49 vs. 58.53, p<0.05, d = 0.67), and children with only ADHD were reported to have a relatively lower mean score in the psychosocial summary scale than children without any chronic condition (46.57 vs. 53.86, p<0.05, d = 1.17). As noted in Table 2, in the subgroup children with migraine/severe headache all 12 scale scores were lower (p<0.05) compared to the subgroup children without any chronic condition, particularly regarding bodily pain (75.84 vs. 88.85, p<0.05, d = 0.62). The lowest mean score for children with asthma was observed for the general health scale (77.30 vs. 90.47, p<0.05, d = 0.77); and the lowest mean score for children with ADHD was observed for the behavior scale (53.90 vs. 73.44, p<0.05, d = 1.21). Across all the five conditions, and in particular for children with ADHD and children with migraine/severe headache, lower mean scale scores were observed for the parent-family specific scales (parental impact-emotional, family activities and family cohesion). S2 Fig shows that the pattern of impairments on HRQOL varies across the five prespecified chronic conditions.

**Table 2:** CHQ-PF28 scores of children with one condition and of children without any reported chronic conditions (n = 5301).

	No chronic condition	Asthma		Eczema		Dyslexia		ADHD		Migraine/severe headache	
	(n = 4539)	(n = 235)		(n = 192)		(n = 207)		(n = 51)		(n = 77)	
	mean score (SD)	mean score (SD)	effect size	mean score (SD)	effect size	mean score (SD)	effect size	mean score (SD)	effect size	mean score (SD)	Effect size
**CHQ-PF28 Summary scales**											
Physical Component Summary Component Scale	58.53 (4.28)	54.49* (6.01)	0.67^b^ *	56.75* (5.40)	0.33^a^ *	58.53 (4.90)	0,00	59.93 * (5.13)	-0.27^a^*	54.89* (6.68)	0.55^b^*
Psychosocial Component Summary Component Scale	53.86 (5.87)	53.72 (5.89)	0.02	52.66* (6.37)	0.19 *	51.51* (6.23)	0.38^a^ *	46.57 * (6.21)	1.17^c^ *	49.72* (8.69)	0.48^a^*
**CHQ-PF28 Child scales**											
Physical Functioning	98.44 (6.51)	93.14* (11.97)	0.44^a^*	96.70* (8.20)	0.21^a^ *	96.99* (9.63)	0.15 *	98.69 (5.73)	-0.04	94.81 * (9.97)	0.36^a^ *
Role/Social Emotional Behavioral	98.80 (6.58)	97.59 (12.28)	0.10	97.05* (11.69)	0.15 *	95.17* (12.65)	0.29^a^*	96.73* (10.01)	0.21^a^*	92.21* (18.65)	0.35^a^ *
Role/Social-Physical	98.84 (6.65)	97.31* (11.42)	0.13 *	97.57 (9.94)	0.13	97.91 (8.11)	0.12	97.39 (14.67)	0.10	95.24* (12.93)	0.28^a^ *
Bodily Pain	88.85 (15.99)	84.51* (17.98)	0.24^a^ *	82.19* (17.41)	0.38^a^ *	87.73 (15.46)	0.07	89.41 (14.06)	-0.04	75.84* (20.86)	0.62^b^ *
Behavior	73.44 (14.04)	72.91 (13.84)	0.04	70.66 (14.76)	0.19 *	69.43 (13.50)	0.29^a^ *	53.90 (16.15)	1.21^c^ *	67.94 (16.99)	0.32^a^*
Mental Health	83.19 (13.54)	82.62 (13.87)	0.04	82.03 (12.64)	0.09	81.04 (13.33)	0.16 *	73.37 (13.64)	0.72^b^ *	73.92 (17.75)	0.52^b^*
Self-Esteem	82.38 (12.64)	80.60 (11.86)	0.14 *	79.45 (12.03)	0.23^a^*	77.54 (12.65)	0.38^a^ *	75.90 (11.94)	0.51^b^ *	77.65 (12.52)	0.38^a^*
General Health Perception	90.47 (12.17)	77.30 (17.05)	0.77^b^*	86.69 (14.19)	0.27^a^*	90.92 (11.72)	-0.04	88.16 (14.36)	0.16	82.19 (16.01)	0.52^b^*
**CHQ-PF28 Parent and Family Impact scales**											
Parental Impact-Emotional	92.96 (11.22)	89.10 (13.63)	0.28^a^*	89.52 (13.42)	0.26^a^*	89.55 (12.33)	0.26^a^*	85.29 (13.39)	0.57^b^*	85.23 (17.88)	0.43^a^*
Parental Impact-Time	97.39 (10.38)	96.60 (12.14)	0.07	96.79 (10.36)	0.06	96.78 (10.14)	0.06	90.85 (22.44)	0.29^a^*	93.07 (16.96)	0.25^a^*
Family Activities	93.47 (12.26)	92.71 (14.37)	0.05	90.30 (15.33)	0.21^a^*	94.38 (10.71)	-0.07	79.17 (23.67)	0.60^b^*	86.69 (18.06)	0.38^a^*
Family Cohesion	80.84 (17.01)	82.47 (16.87)	-0.10	76.82 (17.50)	0.23^a^*	80.39 (17.04)	0.03	71.76 (16.52)	0.53^b^*	75.84 (16.94)	0.29^a^ *

SD: standard deviation

* P <0.05

Effect size (d)

a means small difference when 0.2≤d<0.5 small difference

b means moderate difference when 0.5≤d<0.8

c means large difference when d≥0.8; for others that d was less than 0.2, we didn’t mark them in our table.

Adjusting for potential confounders, the same pattern of significant differences was observed using General Linear Models (see Table 3).

**Table 3:** Difference in scale scores and summary scale scores between children with one of five common childhood conditions/disorders and children without reported chronic condition by General Linear Models when multiple conditions were excluded (n = 5301).

		PF	REB	RF	BP	BE	MH	SE	GH	PE	PT	FA	FC	PhS	PsS
**Asthma vs. No chronic condition**	B	-5.34*	-0.19	-1.30*	-2.31	1.07	0.35	-0.76	-12.19*	-1.31	-0.18	0.68	1.67	-3.72*	0.74
	[95%CI]	[-6.41, -4.26]	[-1.41, 1.03]	[-2.46, -0.14]	[-4.65, 0.02]	[-1.11, 3.26]	[-1.73, 2.44]	[-2.72, 1.19]	[-14.10, -10.28]	[-0.36, 0.44]	[-1.79, 1.43]	[-1.24, 2.60]	[-0.96, 4.30]	[-4.39, -3.05]	[-0.18, 1.66]
**Eczema vs. No chronic condition**	B	-1.52*	-1.85*	-1.12	-4.34*	-2.42*	-0.65	-3.35*	-2.82*	-2.32*	-0.09	-2.13*	-4.17*	-1.22*	-1.08*
	[95%CI]	[-2.64, -0.39]	[-3.13, -0.58]	[-2.34, 0.10]	[-6.79, -1.90]	[-4.70, -0.12]	[-2.84, 1.53]	[-5.40, -1.30]	[-4.83, -0.82]	[-4.16, -0.48]	[-1.78, 1.60]	[-4.14, -0.12]	[-6.62, -1.41]	[-1.93, -0.52]	[-2.04, -0.12]
**Dyslexia vs. No chronic condition**	B	-1.22*	-3.09*	-0.71	-0.71	-4.41*	-2.09	-3.72*	0.42	-4.43*	-1.11	-0.57	-0.96	0.07	-2.36*
	[95%CI]	[-2.38, -0.07]	[-4.41, -1.78]	[-3.22, 1.80]	[-3.22, 1.80]	[-6.76, -2.06]	[-4.33, 0.15]	[-5.82, -1.62]	[-1.64, 2.47]	[-6.32, -2.54]	[-2.85, 0.62]	[-2.63, 1.50]	[-1.88, 3.79]	[-0.66, 0.79]	[-3.35, -1.37]
**ADHD vs. No chronic condition**	B	1.02	-2.15	1.78	2.41	-19.51*	-8.34*	-6.26*	-0.36	-7.56*	-5.18*	-11.96*	-8.24*	2.67*	-7.14*
	[95%CI]	[-1.24, 3.27]	[-4.70, 0.40]	[-0.65, 4.21]	[-2.47, 7.30]	[-24.11, -14.97]	[-12.70, -3.99]	[-10.34, -2.17]	[-4.35, 3.64]	[-11.23, -3.89]	[-8.55, -1.80]	[-15.97, -7.94]	[-13.75, -2.73]	[1.27, 4.08]	[-9.06, -5.21]
**Migraine/severe headache vs. No chronic condition**	B	-2.31*	-7.83*	-2.44*	-8.15*	-4.69*	-5.94*	-2.24	-6.38*	-5.08*	-5.09*	-4.41*	-4.83*	-2.54*	-3.30*
	[95%CI]	[-4.18, -0.44]	[-9.95, -5.71]	[-4.46, -0.42]	[-12.20, -4.09]	[-8.48, -0.90]	[-9.56, -2.33]	[-5.63, 1.15]	[-9.70, -3.06]	[-8.13, -2.03]	[-7.89, -2.29]	[-7.75, -1.08]	[-9.40, -0.26]	[-3.70, -1.37]	[-4.90, -1.70]

Children’s age, gender, ethnic background, body mass index, single parent family, number of acute health complaints and paternal educational level were considered as potential confounders in General Linear Models.

* P<0.05.

PhS Physical Summary Component Scale; PsS Psychosocial Summary Component Scale; PF physical functioning; REB role functioning: emotional/behavior; RF role functioning: physical; BP bodily pain and discomfort; BE general behavior; MH mental health; SE self-esteem; GH general health perceptions; PE parental impact: emotional; PT parental impact: time; FA family activities; FC family cohesion.

Percentages of children with multiple chronic conditions are presented in S1 Table. When children with multiple chronic conditions were taken into account, the mean scores of CHQ-PF28 summary and profile scales were generally lower (see Supplementary S2 Table) compared to children with only one of the five prevalent chronic conditions. But patterns of differences between children with and without specific chronic conditions are similar.

Gender-specific differences in CHQ-PF28 scales between children with only one chronic condition and children with no chronic condition are presented in S3 Table. Compared to girls with no chronic condition, girls with asthma had significantly lower scores in Mental Health Scale, while the difference between boys with asthma and boys with no chronic condition was not significant. Girls with eczema had significantly lower scores in Psychosocial Component Summary Scale, Behavior, Self Esteem, Family Activities and Family Cohesion scales than girls with no chronic condition, while these differences between boys with eczema and boys with no chronic condition were not significant. The patterns were almost the same in girls and boys with dyslexia. Girls with ADHD were reported with significantly higher score in the Physical Component Summary Scale, Physical Functioning and Bodily Pain scales than girls with no chronic condition, while impacts of ADHD on boys in psychology/behavior-related scales (i.e. Psychosocial Component Summary Scale, Behavior, Mental Health, Self Esteem scales) were stronger than on girls. Patterns were found to vary across boys and girls with migraine/severe headache.

Regarding children with asthma, mean scores of the physical summary scale, physical functioning scale, role functioning-physical scale and general health perception scale were significantly lower in children who were seen by the family physician or medical specialist compared to children who were not (see S4 Table). Children with dyslexia who were seen by the family physicians or medical specialists were presented with somewhat higher scores of the parental impact-emotional scale than children did not.

## Discussion

The present study demonstrates lower HRQOL scores of children with a prevalent chronic condition (asthma, eczema, dyslexia, ADHD, or migraine/severe headache) compared with children without any chronic condition. The pattern of impaired HRQOL is specific across the prespecified conditions, which is consistent with clinical benchmarks reported in the CHQ Manual [31]. When comorbidity is taken into account, the HRQOL of children is generally lower than when children with comorbidity were excluded from the analysis.

### Asthma

Current analyses revealed that children with asthma more often lived in the single parent family compared to children without chronic conditions, which is consistent with an early study regarding association of family structure and the prevalence of asthma [36]. As reported by parents, the greatest impact of asthma is observed for ‘physical’ aspects of HRQOL such as physical summary scale, physical functioning, and bodily pain. This observation is consistent with previously reported findings [9, 37]. Parents of children with asthma perceived their child’s health as relatively poor and likely to get worse. As noted by others, in our study, significant difference regarding self-esteem and mental health were not observed [38], for which we have no explanation.

### Eczema

An association in the present investigation was shown between the presence of eczema and the family structure, which consists with a previous study [39]. ‘Physical’ and ‘psychosocial’ aspects of HRQOL were affected by the presence of eczema, which is consistent with prior research [7]. Significantly lower scale scores were observed for physical functioning and bodily pain relative to children without any chronic condition. This observation could be explained in part by the most prevalent symptoms of eczema: itching and soreness [7, 40], which may limit children in their activities and in playing sports. Impaired self-esteem was observed in the present study. It has been suggested that children with eczema may experience comments regarding their appearance, teasing, bullying or even peer rejection, leading to embarrassments and lack of confidence[10, 40]. A gender-specific difference was observed. Girls were disturbed more than boys regarding overall psychosocial HRQOL, behavior and self-esteem. Perhaps the visible redness, inflamed and scaly rashes may causes more stress in girls than in boys, because in general, socially constructed notions require girls to be attractive in appearance.

### Dyslexia

Children with dyslexia in the present study were more often Dutch in the investigation. It is consistent with findings from DHIS 2009–2015 that showed fewer cases dyslexia in children with a western background (Dutch vs. western: 9% vs. 7%) and with a non-western background (Dutch vs. non-western: 9% vs. 2%) [41]. A possible explanation is that for children with a migration background in the Netherlands, the Dutch language may not be their primary language. Their multilingual upbringing may hamper the timely diagnosis of dyslexia, as reading problems could be mistaken for an overall struggle in learning the Dutch language. The most notable observations for children with dyslexia were on the ‘psychosocial’ aspects of HRQOL, including the CHQ Psychosocial Summary Component, the role functioning-behavior/emotional scale, general behavior and self-esteem. This may be due in part to the manifestations of dyslexia, which are characterized by difficulties in reading, and/or spelling, listening, writing. Children with dyslexia may be struggling with schoolwork and may feel inferior to their peers [42]. Data on HRQOL of dyslexia children is rare in both clinical and population studies.

### ADHD

A higher score on the Physical Summary Component scale (PhS) was observed for children with ADHD compared to children without any chronic condition. It is possible that children with restless behavior and other aspects of ADHD excel in the ‘physical’ aspects of health given the very ‘physical’ nature of their condition and in direct response to the pronounced deficits on the more ‘psychosocial’ component. Thus, not surprising, lower scores were observed for general behavior, mental health and self-esteem for children with ADHD. Additionally, the HRQOL of parents and families were significantly impacted. These findings are inconsistent with others’ previous work [31, 43, 44]. Particularly, these impacts were stronger in boys than in girls. Current analyses revealed that parents reported higher scores in physical component summary, physical functioning and bodily pain than children with no chronic condition. It should be taken cautiously considering the very small sample size of girls and boys with ADHD in the present analyses.

### Migraine/Severe headache

Current analyses revealed that parents of children with migraine/severe headache had lower education level than parents of children without chronic condition, which is consistent with Bugdayci et al. who showed that low education of mothers was significantly associated with the presence of headache in children [45]. Some studies indicated that low economic status of family (income) may be a risk factor of presence of migraine/headache [45–47]. Education is often correlated with income status. Parents of children with migraine/severe headache reported lower scores for almost all CHQ-PF28 scales than children without any chronic condition. Their impaired ‘physical’ HRQOL might be explained by the physically painful nature of this condition. But low scores on mental health and family aspects of HRQOL suggest that the burden of this condition is also psychosocial in nature as well. Recurrent migraine/severe headaches may impact on the school performances and may limit social activities with peers, and may decreased home/family activities[48].

In reality, it is not uncommon for children to have more than one chronic condition. The present study shows that children, who had additional chronic conditions except for one of the five most prevalent conditions, generally had lower HRQOL compared to children with only one specific condition. These results are consistent with findings in two population-based studies, which reported poorer HRQOL of children with more than one chronic condition but did not explore the specific burden of chronic conditions on children’s HRQOL [19, 20].

Regarding the mean score of the parental impact-emotional scale in children with dyslexia, those who were seen by the family physicians or medical specialists presented a higher score than children who did not. It might be explained by a positive treatment effect and consequently, relief with regard to the negative impact of dyslexia on parental emotions.

Linking HRQOL data in children with chronic conditions to appropriate interventions to improve HRQOL outcomes has not yet been empirically demonstrated in pediatrics [49]. However, adult studies and some pediatric trials have indicated the potential value of application of the standard HRQOL measurement in practice and research [49–52]. In addition to being of benefit to clinicians and patients, HRQOL may also be an important markers for health policy makers and payment systems to identify those “at risk” for greater need of health care services and subsequently, interventions targeted to more specific domains of impairment [50].

### Strengths and limitations

There are several strengths to this study. Namely, the large population-based sample allowed us to compare HRQOL of children with regard to prevalent chronic condition(s). There have been studies assessing the impact of individual chronic conditions of children on HRQOL [6, 9, 12, 43, 53], however studies comparing HRQOL profiles across different prevalent chronic conditions compared with HRQOL of children without any conditions are scarce [20]. Second, HRQOL was evaluated using the CHQ-PF28, a widely regarded and comprehensive general measure that allowed for the assessment of both the ‘psychosocial’ as well as the ‘physical’ burden of these conditions on the child and his/her parent and family. Third, the present analyses adjusted for potential confounders on the associations between the presence of a chronic condition and HRQOL.

There are several limitations that should be noted. First, ‘causation’ could not be evaluated due to the cross-sectional methodology employed for this study. Second, the CHQ-PF28 is a parent proxy measure. Limitations in study design precluded use of the child self-report version (CHQ-CF87) in concert with the parent version. Thus, data presented herein are from the parents’ perspective and ‘burden profiles’ may differ from children’s point of view and may also differ by age and gender. The discordance between parent-report and child self-report has been noted in previous research [54–57]. It is known, for example, that particularly for mental disorders, parents may underestimate the impact on child’s school experience and social functioning whereas children tend to estimate their HRQOL similar to their peers [13, 43, 58]. Thus, further work to better understand the unique ‘burden’ on HRQOL across common childhood conditions from the child perspective is warranted.

## Conclusion

Prevalent chronic conditions during childhood may place a burden on HRQOL of school-aged children, parents and family, however, little is known regarding the exact profiles of burden of the most prevalent chronic conditions on HRQOL in school-aged children, especially at a large-population level. The present study contributes to fill in the gap by illustrating the specific HRQOL profiles impacted by prevalent chronic conditions in a representative, national sample of school-aged children. These specific HRQOL profiles will help paediatricians and children public health professionals to understand the multidimensional impact of these specific chronic conditions on the HRQOL of children, parents and families. What´s more, the present study has provided the national reference values of HRQOL of school-aged children, which could be used for comparison of HRQOL between studies.

## Supporting information

S1 Fig Flow chart of the population for analysis (N = 5301). (PDF)

S2 Fig Differences in the mean scores on the CHQ-PF28 scales between subgroups of children with a condition (asthma, eczema, ADHD, dyslexia, migraine/severe headache) and children with no reported chronic conditions (n = 5301). (PDF)

S1 Table Comorbidity of children with asthma, eczema, ADHD, dyslexia and migraine. (DOCX)

S2 Table CHQ-PF28 scores of children with one or more condition and of children without any reported chronic condition. (DOCX)

S3 Table Gender-specific difference in CHQ-PF28scores between children with one condition and children without any chronic conditions (n = 5301). (DOCX)

S4 Table Differences in the mean scores of CHQ-PF28 scales and summaries between children who were seen by the family physicians or medical specialist and who were not. (DOCX)

## References

[1] Van Cleave J, Gortmaker SL, Perrin JM. DYnamics of obesity and chronic health conditions among children and youth. JAMA. 2010;303(7):623–30. doi: 10.1001/jama.2010.104 20159870 10.1001/jama.2010.104

[2] Perrin JM, Bloom SR, Gortmaker SL. The increase of childhood chronic conditions in the United States. Jama. 2007;297(24):2755–9. doi: 10.1001/jama.297.24.2755 17595277 10.1001/jama.297.24.2755

[3] Akinbami LJ, Moorman JE, Garbe PL, Sondik EJ. Status of childhood asthma in the United States, 1980–2007. Pediatrics. 2009;123 Suppl 3:S131–45. 19221156 10.1542/peds.2008-2233C

[4] Robison LM, Sclar DA, Skaer TL, Galin RS. National trends in the prevalence of attention-deficit/hyperactivity disorder and the prescribing of methylphenidate among school-age children: 1990–1995. Clin Pediatr (Phila). 1999;38(4):209–17. 10326176 10.1177/000992289903800402

[5] Pastor PN, Reuben CA. Diagnosed attention deficit hyperactivity disorder and learning disability: United States, 2004–2006. Vital Health Stat 10 2008;(237):1–14. 18998276

[6] Clarke SA, Eiser C. The measurement of health-related quality of life (QOL) in paediatric clinical trials: a systematic review. Health Qual Life Outcomes. 2004;2:66 doi: 10.1186/1477-7525-2-66 15555077 10.1186/1477-7525-2-66 PMC534785

[7] Beattie PE, Lewis-Jones MS. A comparative study of impairment of quality of life in children with skin disease and children with other chronic childhood diseases. Br J Dermatol. 2006;155(1):145–51. doi: 10.1111/j.1365-2133.2006.07185.x 16792766 10.1111/j.1365-2133.2006.07185.x

[8] Juniper EF, Guyatt GH, Feeny DH, Ferrie PJ, Griffith LE, Townsend M. Measuring quality of life in children with asthma. Qual Life Res. 1996;5(1):35–46. 8901365 10.1007/BF00435967

[9] Merikallio VJ, Mustalahti K, Remes ST, Valovirta EJ, Kaila M. Comparison of quality of life between asthmatic and healthy school children. Pediatric Allergy and Immunology. 2005;16(4):332–40. doi: 10.1111/j.1399-3038.2005.00286.x 15943597 10.1111/j.1399-3038.2005.00286.x

[10] Lewis-Jones S. Quality of life and childhood atopic dermatitis: the misery of living with childhood eczema. Int J Clin Pract. 2006;60(8):984–92. doi: 10.1111/j.1742-1241.2006.01047.x 16893440 10.1111/j.1742-1241.2006.01047.x

[11] Hafkamp-de Groen E, Raat H. Asthma and Health Related Quality of Life in Childhood and Adolescence: INTECH Open Access Publisher; 2012.

[12] Klassen AF, Miller A, Fine S. Health-related quality of life in children and adolescents who have a diagnosis of attention-deficit/hyperactivity disorder. Pediatrics. 2004;114(5):e541–7. doi: 10.1542/peds.2004-0844 15520087 10.1542/peds.2004-0844

[13] Pongwilairat K, Louthrenoo O, Charnsil C, Witoonchart C. Quality of life of children with attention-deficit/hyper activity disorder. J Med Assoc Thai. 2005;88(8):1062–6. 16404833

[14] Froisland DH, Graue M, Markestad T, Skrivarhaug T, Wentzel-Larsen T, Dahl-Jorgensen K. Health-related quality of life among Norwegian children and adolescents with type 1 diabetes on intensive insulin treatment: a population-based study. Acta Paediatr. 2013;102(9):889–95. doi: 10.1111/apa.12312 23738648 10.1111/apa.12312

[15] Petersen S, Hagglof BL, Bergstrom EI. Impaired health-related quality of life in children with recurrent pain. Pediatrics. 2009;124(4):e759–67. doi: 10.1542/peds.2008-1546 19736269 10.1542/peds.2008-1546

[16] Schipper H, Clinch J, Olweny C. (1996) Quality of Life Studies: Definitions and Conceptual Issues.New York: Lippincott-Raven

[17] Hofman A, Jaddoe VWV, Mackenbach JP, Moll HA, Snijders RFM, Steegers EAP, et al Growth, development and health from early fetal life until young adulthood: the Generation R Study. Paediatric and perinatal epidemiology. 2004;18(1):61–72. 14738548 10.1111/j.1365-3016.2003.00521.x

[18] Ravens-Sieberer U, Wille N, Erhart M, Bettge S, Wittchen H-U, Rothenberger A, et al Prevalence of mental health problems among children and adolescents in Germany: results of the BELLA study within the National Health Interview and Examination Survey. Eur Child Adolesc Psychiatry. 2008;17(1):22–33. 19132301 10.1007/s00787-008-1003-2

[19] Waters E, Davis E, Nicolas C, Wake M, Lo SK. The impact of childhood conditions and concurrent morbidities on child health and well-being. Child Care Health Dev. 2008;34(4):418–29. doi: 10.1111/j.1365-2214.2008.00825.x 19154551 10.1111/j.1365-2214.2008.00825.x

[20] Lee SL, Cheung YF, Wong HS, Leung TH, Lam TH, Lau YL. Chronic health problems and health-related quality of life in Chinese children and adolescents: a population-based study in Hong Kong. BMJ Open. 2013;3(1). 10.1136/bmjopen-2012-001183 PMC3549227 23293240

[21] Sawyer MG, Whaites L, Rey JM, Hazell PL, Graetz BW, Baghurst P. Health-related quality of life of children and adolescents with mental disorders. J Am Acad Child Adolesc Psychiatry. 2002;41(5):530–7. doi: 10.1097/00004583-200205000-00010 12014785 10.1097/00004583-200205000-00010

[22] Sawyer MG, Reynolds KE, Couper JJ, French DJ, Kennedy D, Martin J, et al Health-related quality of life of children and adolescents with chronic illness—a two year prospective study. Qual Life Res. 2004;13(7):1309–19. doi: 10.1023/B:QURE.0000037489.41344.b2 15473509 10.1023/B:QURE.0000037489.41344.b2

[23] Houben-van Herten M, Bai G, Hafkamp E, Landgraf JM, Raat H. Determinants of health-related quality of life in school-aged children: a general population study in the Netherlands. PLoS One. 2015;10(5):e0125083 doi: 10.1371/journal.pone.0125083 25933361 10.1371/journal.pone.0125083 PMC4416795

[24] Banning R, Camstra A, Knottnerus P. Sampling theory: Sampling design and estimation methods. The Hague/Heerlen: Statistics Netherlands 2012.

[25] Statistics Netherlands, CBS (internet). Gezondheidsmetingen kinderen: 2001–2013. http://statline.cbs.nl/Statweb/publication/?DM=SLNL&PA=81174NED&D1=7,9,18&D2=0&D3=2&D4=0&D5=a&VW=T

[26] Statistics Netherlands, CBS (internet). Gezondheid, aandoeningen, beperkingen; leeftijd en geslacht, 2010–2013. http://statline.cbs.nl/Statweb/publication/?DM=SLNL&PA=81174NED&D1=7,9,18&D2=0&D3=2&D4=0&D5=a&VW=T

[27] Statistics Netherlands, CBS (internet). Health, disorders, limitations; sex and age, 2010–2013.http://statline.cbs.nl/Statweb/publication/?VW=T&DM=SLEN&PA=81174ENG&D1=2-7,9-11,17-18,20-21,23,26&D2=0&D3=2&D4=a&D5=a&HD=160616-1613&LA=EN&HDR=T&STB=G1,G2,G3,G4.

[28] Raat H, Bonsel GJ, Essink-Bot ML, Landgraf JM, Gemke RJ. Reliability and validity of comprehensive health status measures in children: The Child Health Questionnaire in relation to the Health Utilities Index. J Clin Epidemiol. 2002;55(1):67–76. 11781124 10.1016/s0895-4356(01)00411-5

[29] Raat H, Botterweck AM, Landgraf JM, Hoogeveen WC, Essink-Bot ML. Reliability and validity of the short form of the child health questionnaire for parents (CHQ-PF28) in large random school based and general population samples. J Epidemiol Community Health. 2005;59(1):75–82. doi: 10.1136/jech.2003.012914 15598731 10.1136/jech.2003.012914 PMC1763365

[30] Raat H, Landgraf JM, Bonsel GJ, Gemke R, Essink-Bot M-L. Reliability and validity of the child health questionnaire-child form (CHQ-CF87) in a Dutch adolescent population. Qual Life Res. 2002;11(6):575–81. 12206578 10.1023/a:1016393311799

[31] Landgraf JM, Abetz L, Ware JE. Child Health Questionnaire (CHQ): A User's Manual. 1999 Boston, MA: HealthAct.

[32] Questionnaires Health Survey from 2010 to 2013. Stastistics Netherlands, CBS (internet). https://www.cbs.nl/nl-nl/onze-diensten/methoden/onderzoeksomschrijvingen/aanvullende%20onderzoeksbeschrijvingen/vragenlijsten-gezondheidsenquete-2010-t-m-2013

[33] Schaart R, Mies Moens B, Westerman S. The Dutch Standard Classification of Education,SOI 2006. Published in 2008 Statistics Netherlands Voorburg/Heerlen.

[34] Alders M. Classification of the population with a foreign background in the Netherlands.

[35] Cohen J. Statistical Power Analysis for the Behavioral Sciences. 2nd edn. Hillsdale, New Jersey: L. Erlbaum; 1988.

[36] Matthews A, Haas DM, O'Mathuna DP, Dowswell T. Interventions for nausea and vomiting in early pregnancy. Cochrane Database Syst Rev. 2015;(9):CD007575 doi: 10.1002/14651858.CD007575.pub4 26348534 10.1002/14651858.CD007575.pub4 PMC7196889

[37] Sawyer MG, Spurrier N, Whaites L, Kennedy D, Martin AJ, Baghurst P. The relationship between asthma severity, family functioning and the health-related quality of life of children with asthma. Qual Life Res. 2000;9(10):1105–15. 11401043 10.1023/a:1016655511879

[38] Mohangoo AD, de Koning HJ, Mangunkusumo RT, Raat H. Health-Related Quality of Life in Adolescents with Wheezing Attacks. Journal of Adolescent Health. 2007;41(5):464–71. doi: 10.1016/j.jadohealth.2007.06.002 17950166 10.1016/j.jadohealth.2007.06.002

[39] Shaw TE, Currie GP, Koudelka CW, Simpson EL. Eczema prevalence in the United States: data from the 2003 National Survey of Children's Health. Journal of Investigative Dermatology. 2011;131(1):67–73. doi: 10.1038/jid.2010.251 20739951 10.1038/jid.2010.251 PMC3130508

[40] Lewis-Jones MS, Finlay AY. The Children's Dermatology Life Quality Index (CDLQI): initial validation and practical use. Br J Dermatol. 1995;132(6):942–9. 7662573 10.1111/j.1365-2133.1995.tb16953.x

[41] Statistics Netherlands, CBS (internet). Slightly more children diagnosed with dyslexia, 2016.https://www.cbs.nl/en-gb/news/2016/40/slightly-more-children-diagnosed-with-dyslexia

[42] McNulty MA. Dyslexia and the life course. J Learn Disabil. 2003;36(4):363–81. doi: 10.1177/00222194030360040701 15490908 10.1177/00222194030360040701

[43] Danckaerts M, Sonuga-Barke EJ, Banaschewski T, Buitelaar J, Dopfner M, Hollis C, et al The quality of life of children with attention deficit/hyperactivity disorder: a systematic review. Eur Child Adolesc Psychiatry. 2010;19(2):83–105. doi: 10.1007/s00787-009-0046-3 19633992 10.1007/s00787-009-0046-3 PMC3128746

[44] Marques JC, Oliveira JA, Goulardins JB, Nascimento RO, Lima AM, Casella EB. Comparison of child self-reports and parent proxy-reports on quality of life of children with attention deficit hyperactivity disorder. Health Qual Life Outcomes. 2013;11:186 doi: 10.1186/1477-7525-11-186 24180423 10.1186/1477-7525-11-186 PMC3842746

[45] Bugdayci R, Ozge A, Sasmaz T, Kurt AO, Kaleagasi H, Karakelle A, et al Prevalence and factors affecting headache in Turkish schoolchildren. Pediatr Int. 2005;47(3):316–22. doi: 10.1111/j.1442-200x.2005.02051.x 15910458 10.1111/j.1442-200x.2005.02051.x

[46] Sillanpää M, Piekkala P, Kero P. Prevalence of Headache at Preschool Age in An Unselected Child Population. Cephalalgia. 1991;11(5):239–42. doi: 10.1046/j.1468-2982.1991.1105239.x 1773439 10.1046/j.1468-2982.1991.1105239.x

[47] Padilla-Moledo C, Ruiz JR, Castro-Pinero J. Parental educational level and psychological positive health and health complaints in Spanish children and adolescents. Child Care Health Dev. 2016;42(4):534–43. doi: 10.1111/cch.12342 27097753 10.1111/cch.12342

[48] Powers SW, Patton SR, Hommel KA, Hershey AD. Quality of life in childhood migraines: clinical impact and comparison to other chronic illnesses. Pediatrics. 2003;112(1 Pt 1):e1–5. 12837897 10.1542/peds.112.1.e1

[49] Varni JW, Limbers CA. The pediatric quality of life inventory: measuring pediatric health-related quality of life from the perspective of children and their parents. Pediatr Clin North Am. 2009;56(4):843–63. doi: 10.1016/j.pcl.2009.05.016 19660631 10.1016/j.pcl.2009.05.016

[50] Varni JW, Burwinkle TM, Lane MM. Health-related quality of life measurement in pediatric clinical practice: An appraisal and precept for future research and application. Health and Quality of Life Outcomes. 2005;3:34–. doi: 10.1186/1477-7525-3-34 15904527 10.1186/1477-7525-3-34 PMC1156928

[51] Mangione-Smith R, Schonlau M, Chan KS, Keesey J, Rosen M, Louis TA, et al Measuring the effectiveness of a collaborative for quality improvement in pediatric asthma care: does implementing the chronic care model improve processes and outcomes of care? Ambul Pediatr. 2005;5(2):75–82. doi: 10.1367/A04-106R.1 15780018 10.1367/A04-106R.1

[52] Vickers AJ. Statistical considerations for use of composite health-related quality-of-life scores in randomized trials. Qual Life Res. 2004;13(4):717–23. doi: 10.1023/B:QURE.0000021686.47079.0d 15129882 10.1023/b:qure.0000021686.47079.0d PMC2613680

[53] Eiser C, Morse R. A review of measures of quality of life for children with chronic illness. Arch Dis Child. 2001;84(3):205–11. doi: 10.1136/adc.84.3.205 11207164 10.1136/adc.84.3.205 PMC1718699

[54] Burks ML, Brooks EG, Hill VL, Peters JI, Wood PR. Assessing proxy reports: agreement between children with asthma and their caregivers on quality of life. Ann Allergy Asthma Immunol. 2013;111(1):14–9. doi: 10.1016/j.anai.2013.05.008 23806454 10.1016/j.anai.2013.05.008 PMC3744830

[55] Bastiaansen D, Koot HM, Ferdinand RF, Verhulst FC. Quality of life in children with psychiatric disorders: self-, parent, and clinician report. J Am Acad Child Adolesc Psychiatry. 2004;43(2):221–30. doi: 10.1097/00004583-200402000-00019 14726730 10.1097/00004583-200402000-00019

[56] Davis E, Nicolas C, Waters E, Cook K, Gibbs L, Gosch A, et al Parent-proxy and child self-reported health-related quality of life: using qualitative methods to explain the discordance. Qual Life Res. 2007;16(5):863–71. doi: 10.1007/s11136-007-9187-3 17351822 10.1007/s11136-007-9187-3

[57] Varni JW, Limbers CA, Burwinkle TM. Parent proxy-report of their children's health-related quality of life: an analysis of 13,878 parents' reliability and validity across age subgroups using the PedsQL 4.0 Generic Core Scales. Health Qual Life Outcomes. 2007;5:2 doi: 10.1186/1477-7525-5-2 17201923 10.1186/1477-7525-5-2 PMC1769359

[58] Klassen AF, Miller A, Fine S. Agreement between parent and child report of quality of life in children with attention-deficit/hyperactivity disorder. Child Care Health Dev. 2006;32(4):397–406. doi: 10.1111/j.1365-2214.2006.00609.x 16784495 10.1111/j.1365-2214.2006.00609.x

