# Supplementary figures and images for: Childhood chronic conditions and health-related quality of life: Findings from a large population-based study
Source: PLoS One. 2017 Jun 2;12(6):e0178539. doi: 10.1371/journal.pone.0178539 (PMC5456082; doi:10.1371/journal.pone.0178539)

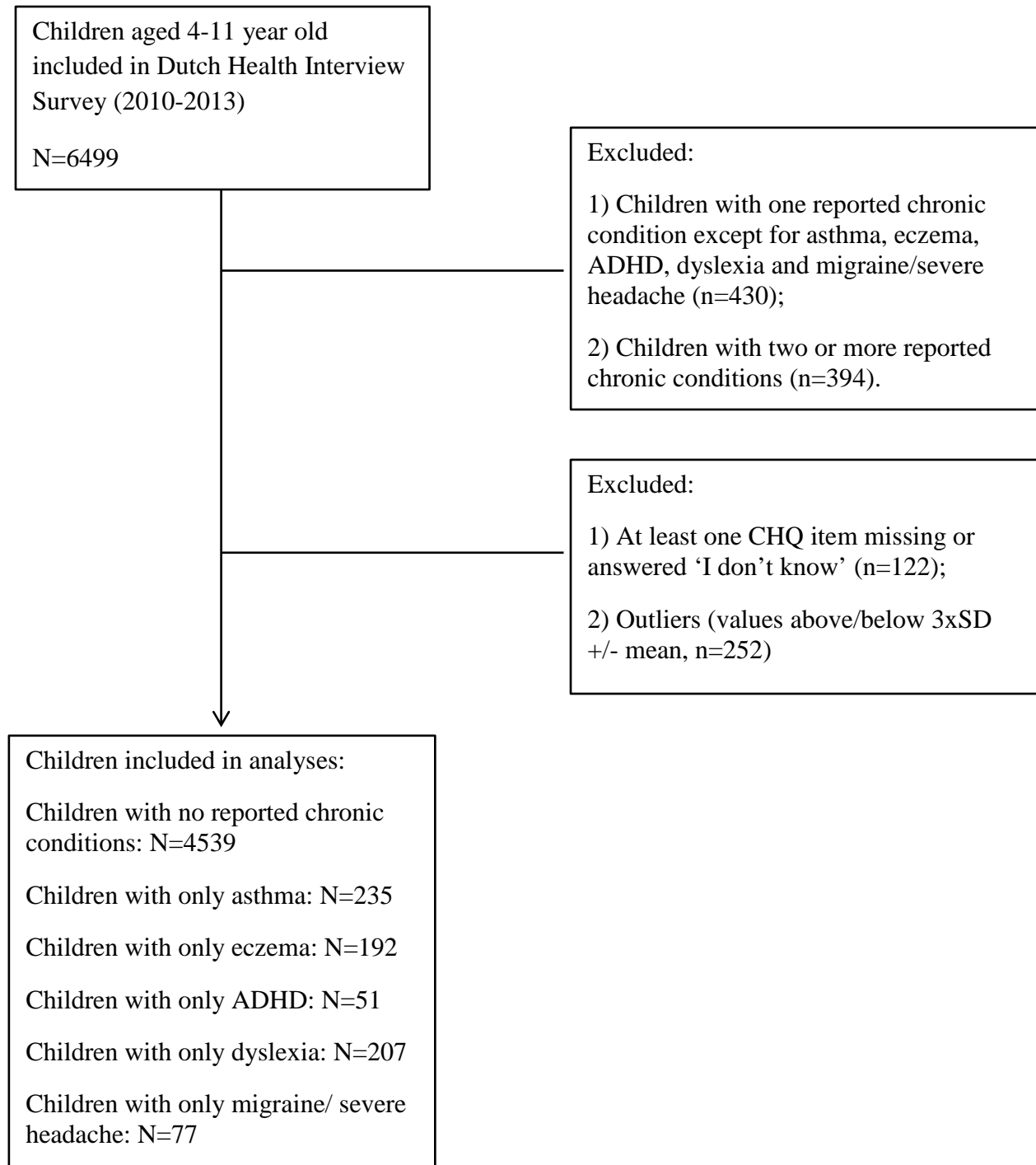

Figure S1. Flow chart of the population for analysis (N=5301)

Supplement: S1 Fig — (PDF) [file pone.0178539.s001.pdf]
