# Supplementary material for: Childhood chronic conditions and health-related quality of life: Findings from a large population-based study
Source: PLoS One. 2017 Jun 2;12(6):e0178539. doi: 10.1371/journal.pone.0178539 (PMC5456082; doi:10.1371/journal.pone.0178539)
Supplement: S2 Fig — (PDF) [file pone.0178539.s002.pdf]

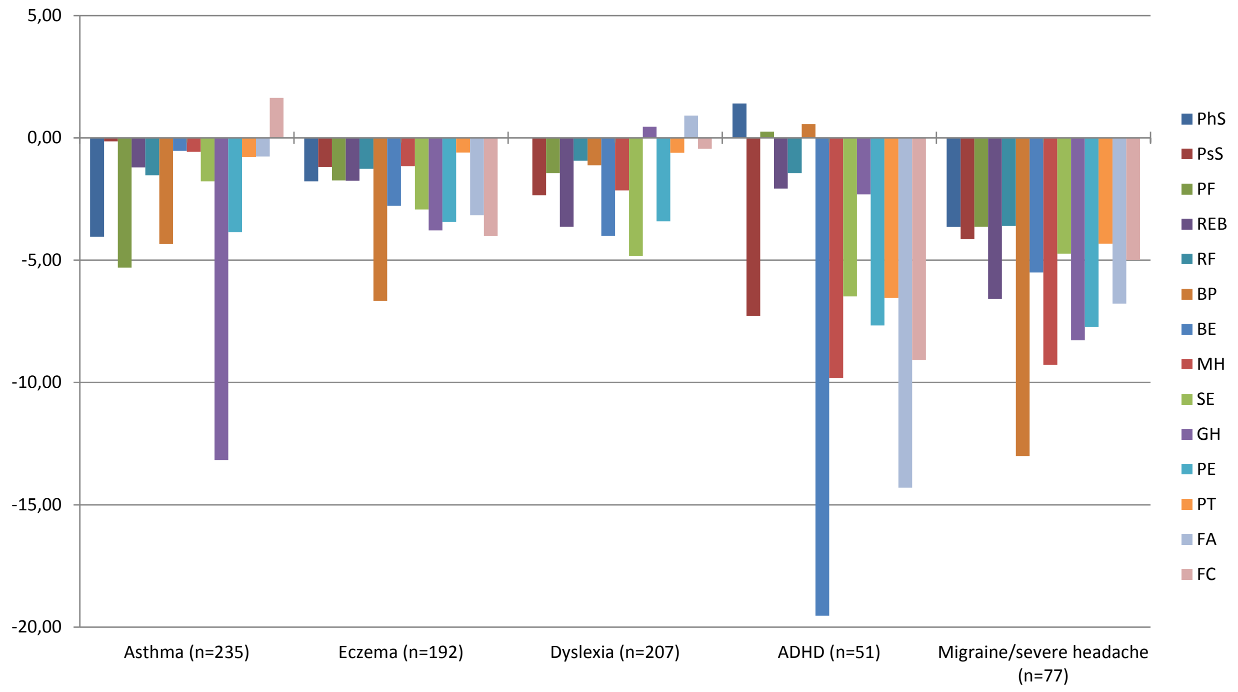

Figure S2. Differences in the mean scores on the CHQ-PF28 scales between subgroups of children with a condition (asthma, eczema, ADHD, dyslexia, migraine/severe headache) and children with no reported chronic conditions (N=5301)

PhS Physical Summary Component Scale; PsS Psychosocial Summary Component Scale; PF physical functioning; REB role functioning-emotional/behavior; RF role functioning-physical; BP bodily pain; BE general behavior; MH mental health; SE self-esteem; GH general health perceptions; PE parental impact: emotional; PT parental impact-time; FA family activities; FC family cohesion; CH change in health
