# Supplementary material for: Childhood chronic conditions and health-related quality of life: Findings from a large population-based study
Source: PLoS One. 2017 Jun 2;12(6):e0178539. doi: 10.1371/journal.pone.0178539 (PMC5456082; doi:10.1371/journal.pone.0178539)
Supplement: S1 Table — (DOCX) [file pone.0178539.s003.docx]

**Table S1. Comorbidity of children with asthma, eczema, ADHD, dyslexia and migraine ^¶^**

|  | **Asthma** | | **Eczema** | | **Dyslexia** | | **ADHD** | | **Migraine/severe headache** | |
| --- | --- | --- | --- | --- | --- | --- | --- | --- | --- | --- |
|  | **(N = 368)** | | **(N = 344)** | | **(N=318)** | | **(N = 140)** | | **(N = 143)** | |
|  | **Valid N** | **%** | **Valid N** | **%** | **Valid N** | | **%** | **%** | **Valid N** | **%** |
| **No comorbidity** | 235 | 63.8 | 192 | 55.8 | 207 | 65.1 | 51 | 36.4 | 77 | 53.8 |
| **Cancer (ever)** | 1 | 0.3 | 0 | 0.0 | 0 | 0.0 | 0 | 0.0 | 0 | 0.0 |
| **Congenital heart disease** | 1 | 0.3 | 6 | 1.7 | 3 | 0.9 | 1 | 0.7 | 1 | 0.7 |
| **Diabetes** | 0 | 0.0 | 1 | 0.3 | 0 | 0.0 | 0 | 0.0 | 0 | 0.0 |
| **Migraine/severe headache** | 18 | 4.9 | 16 | 4.7 | 18 | 5.7 | 8 | 5.7 | . | . |
| **Asthma** | . | . | 69 | 20.1 | 31 | 9.7 | 15 | 10.7 | 18 | 12.6 |
| **Psoriasis** | 1 | 0.3 | 3 | 0.9 | 5 | 1.6 | 0 | 0.0 | 0 | 0.0 |
| **Eczema** | 69 | 18.8 | . | . | 29 | 9.1 | 15 | 10.7 | 16 | 11.2 |
| **Disorders of the intestines** | 8 | 2.2 | 13 | 3.8 | 6 | 1.9 | 7 | 5.0 | 8 | 5.6 |
| **Back disorders** | 0 | 0.0 | 2 | 0.6 | 1 | 0.3 | 0 | 0.0 | 1 | 0.7 |
| **Arthritis/rheumatism** | 0 | 0.0 | 0 | 0.0 | 0 | 0.0 | 0 | 0.0 | 0 | 0.0 |
| **Neck shoulder disorders** | 1 | 0.3 | 1 | 0.3 | 0 | 0.0 | 0 | 0.0 | 2 | 1.4 |
| **Elbow/hand/wrist** | 1 | 0.3 | 1 | 0.3 | 2 | 0.6 | 0 | 0.0 | 1 | 0.7 |
| **Dyslexia** | 31 | 8.4 | 29 | 8.4 | . | . | 16 | 11.4 | 18 | 12.6 |
| **Intellectual disability** | 3 | 0.8 | 2 | 0.6 | 3 | 0.9 | 3 | 2.1 | 0 | 0.0 |
| **ADHD** | 15 | 4.1 | 15 | 4.4 | 16 | 5.0 | . | . | 8 | 5.6 |
| **Other chronic disease not mentioned** | 42 | 11.4 | 53 | 15.1 | 44 | 13.8 | 66 | 47.1 | 25 | 17.5 |

^¶^ because there are overlaps between subgroups of children with asthma, eczema, ADHD, dyslexia, migraine/severe headache, it is not possible to calculate total population for supplementary analyses.
