# Supplementary material for: Childhood chronic conditions and health-related quality of life: Findings from a large population-based study
Source: PLoS One. 2017 Jun 2;12(6):e0178539. doi: 10.1371/journal.pone.0178539 (PMC5456082; doi:10.1371/journal.pone.0178539)
Supplement: S2 Table — (DOCX) [file pone.0178539.s004.docx]

**Table S2. CHQ-PF28 scores of children with one or more condition and of children without any reported chronic condition^¶^**

|  | **No chronic condition** | **Asthma** | | **Eczema** | | **Dyslexia** | | **ADHD** | | **Migraine/severe headache** | |
| --- | --- | --- | --- | --- | --- | --- | --- | --- | --- | --- | --- |
|  | **(n = 4539)** | **(n=368)** | | **(n=344)** | | **(n=318)** | | **(n=140)** | | **(n=143)** | |
|  | **mean score (SD)** | **mean score (SD)** | **effect size** | **mean score (SD)** | **effect size** | **mean score (SD)** | **effect size** | **mean score (SD)** | **effect size** | **mean score (SD)** | **effect**  **size** |
| **CHQ-PF28 Summary scales** | | | | | | | | | | | |
| **Physical Component Summary Component Scale** | 58.53 (4.28) | 53.27 (7.07) | 0.74^b^* | 55.28 (6.71) | 0.49^a^* | 57.66 (6.01) | 0.15* | 57.62 (6.61) | 0.14 | 53.49 (7.19) | 0.70^b^* |
| **Psychosocial Component Summary Component Scale** | 53.86 (5.87) | 52.09 (7.12) | 0.25^a^* | 51.04 (7.52) | 0.37^a^* | 50.14 (7.28) | 0.51^b^* | 44.50 (7.08) | 1.32^c^* | 49.45 (8.05) | 0.55^b^* |
|  | | | | | | | | | | | |
| **CHQ-PF28 Child scales** | | | | | | | | | | | |
| **Physical Functioning** | 98.44 (6.51) | 92.15 (12.25) | 0.51^b^* | 95.48 (9.62) | 0.31^a^* | 96.54 (9.39) | 0.20^a^* | 95.71 (10.71) | 0.25^a^* | 93.40 (11.13) | 0.45^a^* |
| **Role/Social Emotional Behavioral** | 98.80 (6.58) | 94.93 (16.46) | 0.24^a^* | 94.19 (16.05) | 0.29^a^* | 92.14 (17.89) | 0.37^a^* | 87.62 (24.10) | 0.46^a^* | 92.37 (16.85) | 0.36^a^* |
| **Role/Social-Physical** | 98.84 (6.65) | 95.74 (13.36) | 0.23^a^* | 96.22 (12.28) | 0.21^a^ | 96.75 (10.91) | 0.19 | 96.43 (13.67) | 0.18 | 94.64 (12.91) | 0.33^a^* |
| **Bodily Pain** | 88.85 (15.99) | 81.36 (20.65) | 0.36^a^* | 80.64 (20.34) | 0.40^a^* | 86.10 (17.60) | 0.16* | 85.57 (20.65) | 0.16 | 73.29 (21.88) | 0.71^b^* |
| **Behavior** | 73.44 (14.04) | 69.42 (15.92) | 0.25^a^* | 68.38 (16.15) | 0.31^a^* | 67.21 (14.97) | 0.42^a^* | 50.70 (15.06) | 1.51^c^* | 66.28 (16.79) | 0.43^a^* |
| **Mental Health** | 83.19 (13.54) | 80.12 (15.48) | 0.20^a^* | 78.59 (15.03) | 0.31^a^* | 78.93 (14.74) | 0.29^a^* | 72.32 (16.06) | 0.68^b^* | 73.08 (16.77) | 0.60^b^* |
| **Self-Esteem** | 82.38 (12.64) | 79.06 (12.67) | 0.26^a^* | 78.56 (13.13) | 0.29^a^* | 75.73 (13.55) | 0.49^a^* | 73.60 (13.12) | 0.67^b^* | 76.84 (11.73) | 0.44^a^* |
| **General Health Perception** | 90.47 (12.17) | 73.16 (19.78) | 0.88^c^* | 80.22 (20.23) | 0.51^b^* | 86.69 (15.69) | 0.24^a^* | 80.36 (19.04) | 0.53^b^ | 76.80 (17.56) | 0.78^b^* |
|  |  |  |  |  |  |  |  |  |  |  |  |
| **CHQ-PF28 Parent and Family Impact scales** | | | | | | | | | | | |
| **Parental Impact-Emotional** | 92.96 (11.22) | 86.51 (14.60) | 0.44^a^* | 85.79 (15.25) | 0.47^a^* | 87.66 (13.13) | 0.40^a^* | 81.79 (15.68) | 0.71^b^ | 83.04 (18.19) | 0.54^b^* |
| **Parental Impact-Time** | 97.39 (10.38) | 94.61 (15.08) | 0.18* | 94.72 (13.84) | 0.19* | 95.65 (11.29) | 0.15* | 87.86 (22.87) | 0.42^a^ | 94.06 (14.59) | 0.23^a^* |
| **Family Activities** | 93.47 (12.26) | 89.33 (17.19) | 0.24^a^* | 87.83 (17.66) | 0.32^a^* | 91.82 (14.18) | 0.12* | 74.82 (24.50) | 0.76^b^ | 85.58 (18.96) | 0.42^a^* |
| **Family Cohesion** | 80.84 (17.01) | 79.13 (17.86) | 0.10 | 76.53 (18.32) | 0.24^a^* | 77.42 (18.28) | 0.19* | 69.50 (17.27) | 0.66^b^ | 73.78 (18.54) | 0.38^a^* |

SD: standard deviation

*P <0.05

Effect size (d): a means small difference when 0.2≤d<0.5 small difference; b means moderate difference when 0.5≤d<0.8; c means large difference when d≥0.8; for others that d was less than 0.2, we didn’t mark them in our table.

^¶^because there are overlaps between subgroups of children with asthma, eczema, ADHD, dyslexia, migraine/severe headache, it is not possible to calculate total population for supplementary analyses.
