# Supplementary material for: Childhood chronic conditions and health-related quality of life: Findings from a large population-based study
Source: PLoS One. 2017 Jun 2;12(6):e0178539. doi: 10.1371/journal.pone.0178539 (PMC5456082; doi:10.1371/journal.pone.0178539)
Supplement: S3 Table — (DOCX) [file pone.0178539.s005.docx]

**Table S3. Gender-specific difference in CHQ-PF28scores between children with one condition and children without any chronic conditions (n=5301)**

|  | **No chronic condition** | **Asthma** | | | **Eczema** | | **Dyslexia** | | **ADHD** | | **Migraine/severe headache** | |
| --- | --- | --- | --- | --- | --- | --- | --- | --- | --- | --- | --- | --- |
|  | **(Boys n=2242** | **(Boys n=141** | | | **(Boys n=87** | | **(Boys n=118** | | **(Boys n=29** | | **(Boys n=33** | |
|  | **Girls n=2297)** | **Girls n=94)** | | | **Girls n=105)** | | **Girls n=89)** | | **Girls n=22)** | | **Girls n=44)** | |
|  | **mean score** | **mean score** | **effect size** | | **mean score** | **effect size** | **mean score** | **effect size** | **mean score** | **effect size** | **mean score** | **effect size** |
|  | **(SD)** | **(SD)** |  | | **(SD)** |  | **(SD)** |  | **(SD)** |  | **(SD)** |  |
| **CHQ-PF28 Summary Scales** | | | | | | | | | | | | |
| **Physical Summary Component Scale** | | | | | | | | | | | | |
| **Boys** | 58.61 (4.40) | 53.95 (6.39) | 0.73^b^* | | 56.86 (5.39) | 0.32^a^* | 58.86 (4.90) | -0.12 | 59.36 (6.25) | -0.12 | 55.17 (6.63) | 0.52^b^* |
| **Girls** | 58.46 (4.15) | 55.31 (5.31) | 0.59^b^* | | 56.66 (5.42) | 0.33^a^* | 58.10 (4.88) | 0.07 | 60.68 (3.09) | -0.54^b*^ | 54.67 (6.78) | 0.56^b^* |
| **Psychosocial Summary Component Scale** | | | | | | | | | | | | |
| **Boys** | 53.56 (5.81) | 54.16 (6.08) | -0.10 | | 52.62 (6.86) | 0.14 | 51.18 (6.44) | 0.37^a^* | 45.36 (6.81) | 1.20^c^* | 50.21 (8.09) | 0.41^a^* |
| **Girls** | 54.16 (5.91) | 53.06 (5.57) | 0.20 | | 52.70 (5.97) | 0.25^a^* | 51.95 (5.95) | 0.37^a^* | 48.17 (5.03) | 1.01^c^* | 49.36 (9.19) | 0.52^b^* |
|  |  |  |  | |  |  |  |  |  |  |  |  |
| **CHQ-PF28 Child Scales** | | | | | | | | | | | | |
| **Physical Functioning** | | | | | | | | | | | | |
| **Boys** | 98.22 (6.87) | 92.44 (12.41) | 0.47^a^* | | 95.91 (9.30) | 0.25^a^* | 97.18 (9.09) | 0.11 | 97.70 (7.50) | 0.07 | 90.91 (12.25) | 0.60^b^* |
| **Girls** | 98.66 (6.13) | 94.21 (11.26) | 0.40^a^* | | 97.35 (7.15) | 0.18 | 96.75 (10.35) | 0.18 | 100.00 (0.00) | -0.22^a^* | 97.73 (6.60) | 0.14 |
| **Role/Social Emotional Behavioral** | | | | | | | | | | | | |
| **Boys** | 98.81 (6.65) | 97.40 (13.26) | 0.11 | | 96.93 (12.06) | 0.16* | 94.63 (13.05) | 0.32^a^* | 97.70 (8.60) | 0.13 | 93.94 (17.59) | 0.28^a^* |
| **Girls** | 98.80 (6.53) | 97.87 (10.72) | 0.09 | | 97.14 (11.43) | 0.14 | 95.88 (12.12) | 0.24 ^a^* | 95.45 (11.71) | 0.29^a^* | 90.91 (19.51) | 0.40^a^* |
| **Role/Social-Physical** | | | | | | | | | | | | |
| **Boys** | 98.72 (7.07) | 96.22 (13.85) | 0.18 | | 98.08 (9.31) | 0.07 | 98.31 (7.35) | 0.06 | 95.40 (19.36) | 0.17 | 95.96 (11.05) | 0.25^a^* |
| **Girls** | 98.96 (6.21) | 98.94 (5.89) | 0.00 | | 97.14 (10.45) | 0.17 | 97.38 (9.02) | 0.17 | 100.00 (0.00) | -0.17 | 94.70 (14.28) | 0.30^a^* |
| **Bodily Pain** | | | | | | | | | | | | |
| **Boys** | 89.48 (15.69) | 85.53 (16.41) | 0.24^a^* | | 83.68 (16.00) | 0.36^a^* | 88.31 (16.61) | 0.07 | 87.59 (14.55) | 0.12 | 78.18 (16.86) | 0.67^b^* |
| **Girls** | 88.24 (16.25) | 82.98 (20.10) | 0.26^a^* | | 80.95 (18.48) | 0.39^a^* | 86.97 (13.85) | 0.08 | 91.82 (13.32) | -0.22 ^a^* | 74.09 (23.46) | 0.60^b^* |
| **Behavior** | | | | | | | | | | | | |
| **Boys** | 72.03 (14.12) | 72.90 (14.82) | -0.06 | | 70.03 (14.54) | 0.14 | 68.39 (13.15) | 0.26^a^* | 50.00 (14.22) | 1.55^c^* | 65.76 (17.06) | 0.37^a^* |
| **Girls** | 74.82 (13.81) | 72.94 (12.30) | 0.14 | | 71.19 (14.99) | 0.24 ^a^* | 70.80 (13.92) | 0.29^a^* | 59.03 (17.40) | 0.91^c^* | 69.57 (16.96) | 0.31^a^* |
|  |  |  |  | |  |  |  |  |  |  |  |  |
| **Mental Health** | | | | | | | | | | | | |
| **Boys** | 83.31 (13.35) | 84.75 (13.47) | -0.11 | | 83.05 (13.54) | 0.02 | 80.79 (13.93) | 0.18 | 72.13 (13.04) | 0.84^c^* | 72.98 (16.67) | 0.62^b^* |
| **Girls** | 83.08 (13.73) | 79.43 (13.92) | 0.26^a*^ | | 81.19 (11.84) | 0.14 | 81.37 (12.56) | 0.12 | 75.00 (14.55) | 0.56^b^* | 74.62 (18.67) | 0.45^a^* |
| **Self-Esteem** | | | | | | | | | | | | |
| **Boys** | 81.91 (12.26) | 81.44 (12.01) | 0.04 | | 79.31 (11.90) | 0.21^a^ | 76.94 (12.06) | 0.41^a^* | 74.57 (12.42) | 0.59^b^* | 78.41 (12.56) | 0.28^a^* |
| **Girls** | 82.84 (12.98) | 79.34 (11.59) | 0.27 ^a^* | | 79.56 (12.20) | 0.25^a^* | 78.32 (13.42) | 0.34 ^a^* | 77.65 (11.32) | 0.40^a^* | 77.08 (12.60) | 0.44^a^* |
| **General Health Perception** | | | | | | | | | | | | |
| **Boys** | 90.20 (12.42) | 76.58 (17.40) | 0.78^b^* | | 87.01 (14.47) | 0.22^a^* | 90.89 (11.77) | -0.06 | 88.32 (16.16) | 0.12 | 84.05 (15.74) | 0.39^a^* |
| **Girls** | 90.74 (11.92) | 78.38 (16.56) | 0.75^b^* | | 86.43 (14.02) | 0.31^a^* | 90.96 (11.72) | 0.02 | 87.95 (11.93) | 0.23 ^a^* | 80.80 (16.24) | 0.61^b^* |
|  |  |  |  | |  |  |  |  |  |  |  |  |
| **CHQ-PF28 Parent and Family Impact scales** | | | | | | | | | | | | |
| **Parental Impact-Emotional** | | | | | | | | | | | | |
| **Boys** | 92.58 (11.27) | 88.12 (14.66) | | 0.30^a^* | 89.80 (12.14) | 0.23^a^* | 90.36 (11.60) | 0.19* | 83.19 (13.48) | 0.70^b^* | 88.64 (11.84) | 0.33^a^* |
| **Girls** | 93.32 (11.15) | 90.56 (11.84) | | 0.23^a^* | 89.29 (14.44) | 0.28^a^* | 88.48 (13.22) | 0.37 ^a*^ | 88.07 (13.07) | 0.40^a^* | 82.67 (21.09) | 0.51^b^* |
| **Parental Impact-Time** | | | | | | | | | | | | |
| **Boys** | 97.30 (10.29) | 96.22 (12.01) | | 0.09 | 95.59 (12.82) | 0.13 | 96.61 (11.44) | 0.06 | 87.93 (23.10) | 0.41^a^* | 96.97 (9.73) | 0.03 |
| **Girls** | 97.48 (10.47) | 97.16 (12.38) | | 0.03 | 97.78 (7.69) | -0.03 | 97.00 (8.17) | 0.05 | 94.70 (21.45) | -0.13 | 90.15 (20.43) | 0.36^a^* |
| **Family Activities** | | | | | | | | | | | | |
| **Boys** | 93.07 (12.57) | 93.00 (13.96) | | 0.01 | 90.95 (14.60) | 0.15 | 93.86 (11.41) | -0.07 | 75.43 (23.74) | 0.74^b^* | 83.71 (18.08) | 0.52^b^* |
| **Girls** | 93.87 (11.94) | 92.29 (15.02) | | 0.11 | 89.76 (15.96) | 0.26 ^a^* | 95.08 (9.72) | -0.10 | 84.09 (23.20) | 0.42^a^* | 88.92 (17.93) | 0.28 ^a^ |
| **Family Cohesion** | | | | | | | | | | | | |
| **Boys** | 80.26 (16.97) | 83.76 (16.23) | | -0.21^a^* | 76.72 (19.42) | 0.18 | 79.66 (17.96) | 0.03 | 70.17 (17.80) | 0.57^b^* | 78.33 (16.04) | 0.11 |
| **Girls** | 81.40 (17.04) | 80.53 (17.71) | | 0.05 | 76.90 (15.83) | 0.26^a^* | 81.35 (15.79) | 0.00 | 73.86 (14.79) | 0.44^a^* | 73.98 (17.54) | 0.42^a^* |

Effect sizes: a = small difference, b = moderate difference, c =large difference

* Statistically significant difference compared to children with no chronic condition.
