# Supplementary material for: Childhood chronic conditions and health-related quality of life: Findings from a large population-based study
Source: PLoS One. 2017 Jun 2;12(6):e0178539. doi: 10.1371/journal.pone.0178539 (PMC5456082; doi:10.1371/journal.pone.0178539)
Supplement: S4 Table — (DOCX) [file pone.0178539.s006.docx]

**Table S4. Differences in the mean scores of CHQ-PF28 scales and summaries between children who were seen by the family physicians or medical specialist and who were not^&^**

|  | **Asthma** | | | | **Eczema** | | | | **Dyslexia** | | | | **Migraine/severe headache** | | | |  |
| --- | --- | --- | --- | --- | --- | --- | --- | --- | --- | --- | --- | --- | --- | --- | --- | --- | --- |
|  | **Seen by the family physician or medical specialist (n=143)** | | **Not Seen by the family physician or medical specialist (n=92)** | | **Seen by the family physician or medical specialist (n=86)** | | **Not seen by the family physician or medical specialist (n=106)** | | **Seen by the family physician or medical specialist (n=86)** | | **Not seen by the family physician or medical specialist (n=121)** | | **Seen by the family physician or medical specialist (n=28)** | | **Not seen by the family physician or medical specialist (n=49)** | |  |
|  | **mean (SD)** | | **mean (SD)** | | **mean (SD)** | | **mean (SD)** | | **mean (SD)** | | **mean (SD)** | | **mean (SD)** | | **mean (SD)** | |  |
| **CHQ-PF28 Summary Scales** | | | | | | | | | | | | | | | | |  |
| **PhS** | **53.40 (6.52)** | | **56.20 (4.65)** | | 56.78 (5.65) | | 56.73 (5.21) | | 59.07 (4.15) | | 58.15 (5.34) | | 53.20 (8.48) | | 55.85 (5.25) | |  |
| **PsS** | 53.77 (5.54) | | 53.64 (6.43) | | 52.03 (6.88) | | 53.17 (5.91) | | 51.82 (5.95) | | 51.29 (6.44) | | 49.00 (7.72) | | 50.14 (9.25) | |  |
|  |  | |  | |  | |  | |  | |  | |  | |  | |  |
| **CHQ-PF28 Summary Scales** | | | | | | | | | | | | | | | | |  |
| **PF** | **91.67 (12.68)** | | **95.41 (10.44)** | | 96.64 (9.96) | | 96.75 (7.96) | | 97.67 (8.88) | | 96.51 (10.15) | | 92.86 (12.18) | | 95.92 (8.39) | |  |
| **REB** | 97.90 (12.64) | | 97.10 (11.75) | | 97.67 (9.95) | | 96.54 (12.95) | | 94.19 (13.71) | | 95.87 (11.84) | | 91.67 (19.51) | | 92.51 (18.34) | |  |
| **RF** | **96.04 (13.98)** | | **99.28 (4.89)** | | 98.45 (7.06) | | 96.85 (11.75) | | 98.06 (7.84) | | 97.80 (8.31) | | 91.67 (17.27) | | 97.28 (9.22) | |  |
| **BP** | 83.78 (19.57) | | 85.65 (15.21) | | 81.86 (17.52) | | 82.45 (17.39) | | 90.23 (13.28) | | 85.95 (16.66) | | 72.86 (21.92) | | 77.55 (20.26) | |  |
| **BE** | 73.16 (14.52) | | 72.53 (12.77) | | 69.36 (15.36) | | 71.72 (14.24) | | 69.81 (13.44) | | 69.15 (13.60) | | 66.61 (18.53) | | 68.70 (16.20) | |  |
| **MH** | 81.70 (13.68) | | 84.06 (14.12) | | 81.01 (13.44) | | 82.86 (11.94) | | 81.20 (13.90) | | 80.92 (12.97) | | 72.32 (15.56) | | 74.83 (18.98) | |  |
| **SE** | 80.59 (11.40) | | 80.62 (12.62) | | 79.12 (12.60) | | 79.72 (11.60) | | 79.31 (10.66) | | 76.27 (13.79) | | 76.34 (9.15) | | 78.40 (14.12) | |  |
| **GH** | **74.43 (17.08)** | | **81.75 (16.11)** | | 85.77 (14.85) | | 87.44 (13.65) | | 91.51 (10.08) | | 90.50 (12.78) | | 80.27 (15.30) | | 83.29 (16.45) | |  |
|  |  | |  | |  | |  | |  | |  | |  | |  | |  |
| **CHQ-PF28 Summary Scales** | | | | | | | | | | | | | | | | |  |
| **PE** | | 88.72 (13.04) | | 89.67 (14.54) | | 88.23 (15.14) | | 90.56 (11.80) | | **92.00 (9.95)** | | **87.81 (13.55)** | | 84.38 (20.30) | | 85.71 (16.54) | |
| **PT** | | 96.04 (12.34) | | 97.46 (11.83) | | 95.15 (12.49) | | 98.11 (8.07) | | 96.51 (13.06) | | 96.97 (7.45) | | 89.88 (23.72) | | 94.90 (23.72) | |
| **FA** | | 91.96 (14.03) | | 93.89 (14.88) | | 88.23 (16.86) | | 91.98 (13.82) | | 95.35 (8.79) | | 93.70 (11.87) | | 84.37 (18.52) | | 88.01 (17.85) | |
| **FC** | | 83.53 (16.73) | | 80.82 (17.05) | | 75.87 (19.43) | | 77.59 (15.81) | | 80.70 (16.70) | | 80.16 (17.34) | | 79.11 (17.80) | | 73.98 (16.33) | |

^&^data shown in this table is from children with one of the five prevalent chronic conditions (asthma, eczema, dyslexia or migraine/severe headache). The information on ADHD was not collected by the interview. Bold print indicates statistical significance (p<0.05).

PhS Physical Summary Component Scale ; PsS Psychosocial Summary Component Scale; PF physical functioning; REB role functioning: emotional/behavior; RF role functioning: physical; BP bodily pain and discomfort; BE general behavior; MH mental health; SE self-esteem; GH general health perceptions; PE parental impact: emotional; PT parental impact: time; FA family activities; FC family cohesion.
